# Sickle cell disease is a global prototype for integrative research and healthcare

**DOI:** 10.1002/ggn2.10037

**Published:** 2021-02-25

**Authors:** Charmaine D. M. Royal, Michael Babyak, Nirmish Shah, Shantanu Srivatsa, Kearsley A. Stewart, Paula Tanabe, Ambroise Wonkam, Monika Asnani

**Affiliations:** ^1^ Department of African & African American Studies Duke University Durham North Carolina USA; ^2^ Duke Global Health Institute Duke University Durham North Carolina USA; ^3^ Center on Genomics, Race, Identity, Difference Duke University Durham North Carolina USA; ^4^ Department of Psychiatry and Behavioral Sciences Duke University Medical Center Durham North Carolina USA; ^5^ Department of Medicine Duke University School of Medicine Durham North Carolina USA; ^6^ Duke University School of Nursing Durham North Carolina USA; ^7^ Division of Human Genetics, Department of Medicine, Faculty of Health Sciences University of Cape Town Cape Town South Africa; ^8^ Faculty of Medicine and Biomedical Sciences University of Yaoundé 1 Yaoundé Cameroon; ^9^ Caribbean Institute for Health Research ‐ Sickle Cell Unit The University of the West Indies Kingston Jamaica

**Keywords:** global health, gene‐environment interactions, integrative research, sickle cell disease

## Abstract

Differences in health outcomes and treatment responses within and between global populations have been well documented. There is growing recognition of the need to move beyond simple inventories and descriptions of these differences and our linear explanations for them, and gain a better understanding of the multifaceted systems and networks underlying them in order to develop more precise and effective remedies. Typical targets for such integrative research have been common multifactorial diseases. We propose sickle cell disease, one of the most common monogenic diseases, as an ideal candidate for elucidating the complexity of the influences of endogenous and exogenous factors on disease pathophysiology, phenotypic diversity, and variations in responses to treatments at both the individual and population levels. We provide data‐informed representations of diverse contributors to sickle cell disease complications that could guide innovative efforts to advance scientific knowledge, clinical practice, and policy formulation related to the disease; help improve outcomes for people worldwide with sickle cell disease; and inform approaches to studying and addressing other diseases.

## Graphical abstract

Sickle cell disease, one of the most common monogenic diseases with global reach, illustrates the complexity of the influences of endogenous and exogenous factors on disease pathophysiology, phenotypic diversity, and variations in responses to treatments. Multiple domains of influence comprise intricate networks that interact with one another over the life course at both the individual and population levels. These biological, psychological, clinical, behavioral, sociocultural, structural, and environmental variables interact to produce episodes of clinical disease and changing experience of disability and resilience. Data from both clinical records and affected individuals suggest conceptual models that can in turn be tested statistically. Other monogenic disorders could also serve to model and decode more common and etiologically complicated health conditions, advancing global efforts to improve and sustain health.

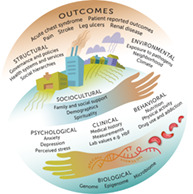

## 1 INTRODUCTION

The moral imperative to improve health and eliminate health inequities and disparities calls for fundamental changes in how disease and individual and population health differences are investigated and addressed.^1^ Integrative (holistic) research and global health are recurring themes in this discourse and in the burgeoning shift toward transdisciplinary, translational, and transformational science.^1^, ^2^ The United States (US) Precision Medicine Initiative (PMI) and other similar country‐ and continent‐wide biomedical efforts have emerged from this shift, heralding a potential global revolution in research and healthcare.^2^, ^3^, ^4^ However, the capacity of these initiatives to effectively facilitate optimal health, disease prevention, and health equity will be largely dependent on their commitment to and embodiment of the integrative guiding frameworks they espouse.

Advances in biology (eg, genomics) are providing new insights into the molecular and other endogenous contributors to health and the etiology, course, and treatment of disease, while epidemiological and health disparities research have been shedding light on social and other exogenous dimensions. For example, observed differences in health and disease indicators within and between countries and populations have been attributed to factors such as genetic variation, geographical location, cultural and psychosocial influences, behaviors, and unequal distributions of power, income, goods, and services.^2^, ^5^, ^6^, ^7^, ^8^ Any breakdown in health represents a nonlinear phenomenon; therefore, it is critical to view the disease process not solely as a collection of individual risk factors, but to model it as a network of relationships with properties not attributable to individual components.

Despite increased recognition of the need for more integrative approaches to biomedical research, a substantial portion of research (including some claiming to be integrative) is still guided by linear, reductionist heuristics. Furthermore, most of the published studies and emerging initiatives on genotype‐environment interactions (broadly defined) and multi‐level systems science instinctively focus on multifactorial or so‐called complex diseases and traits. It has long been acknowledged that despite their monogenic etiology, classical Mendelian diseases, such as cystic fibrosis, phenylketonuria, hemochromatosis, G6PD deficiency, and the hemoglobinopathies are also complex, generally having variable phenotypic expression due to interactions among an array of genetic and non‐genetic factors.^9^, ^10^ Nonetheless, these disorders traditionally have not been targets for systematic genotype‐environment investigations. Monogenic conditions are poised to bring new dimensions to the study of disease complexity over the life course and to our understanding of health differences at the individual, family, and population levels. They provide a unique opportunity to identify diverse modifiers of risk and resilience as they have uniform etiology, detailed phenotyping of affected individuals, and familial clustering.^9^ Naturally, they are also prime candidates for novel genetic tools and technologies such as gene therapy and somatic cell genome editing (eg, CRISPR) that are accelerating the quest for cures.^11^ Overall, monogenic disorders could serve to model and decode more common and etiologically complicated health conditions, advancing global efforts to improve and sustain health.

## 2 THE CASE OF SICKLE CELL DISEASE

Sickle cell disease (SCD) is the first disease whose genetic etiology was defined, and is one of the most common severe monogenic diseases in humans. SCD refers to a group of recessively inherited blood disorders characterized by the predominance of sickle hemoglobin (HbS), the result of a single nucleotide change in the structural gene for the beta unit of hemoglobin (HBB).^12^, ^13^ The HbS variant is considered to have originated in Africa and subsequently increased rapidly in frequency as a result of the substantial protection from severe malarial infection that it provides to heterozygote carriers (sickle cell trait; SCT).^13^, ^14^ The clinical manifestations of SCD result from increased red cell hemolysis, vaso‐occlusion, and accompanying physiologic changes that lead to acute complications, including acute painful episodes (vaso‐occlusive crises; VOC), susceptibility to infection, acute chest syndrome, and stroke, along with chronic pain and organ damage to the spleen, kidneys, brain, and lungs.^12^ These and other complications extend across the life spectrum and reduce life expectancy of people living with SCD by about 30 years compared to their healthy peers.^13^, ^15^, ^16^

SCD affects approximately 20 million people worldwide and is most prevalent in parts of Africa, the Caribbean, the Mediterranean basin, the Middle East, India, and South and Central America.^13^, ^17^ Notwithstanding the diverse populations affected, in some countries, such as the US, Brazil, and Turkey, SCD has long been misconstrued as a “black disease” or a disease of racially marginalized groups, and this racialized notion of the disease is a key element of its distinctive biocultural story.^18^, ^19^, ^20^ Globally, more than 300 000 babies are born annually with SCD, of which at least 75% are in Africa.^13^ SCD remains a leading source of mortality, morbidity, and health disparity, and has been designated as a major global health problem and priority by the World Health Organization (WHO) and United Nations (UN).^21^, ^22^ Recent major national and global initiatives aimed at providing new financial and other resources for monitoring, researching, treating, and curing SCD demonstrate increasing attention to the disease.^11^, ^23^, ^24^, ^25^

The frequency and severity of SCD complications vary markedly between patients and at different ages. Both genetic and non‐genetic factors are known to influence SCD severity.^10^, ^13^, ^26^, ^27^ For example, high fetal hemoglobin (HbF) levels have long been associated with less severity^11^; HbF levels are under genetic control and are amenable to therapeutic manipulation in SCD.^28^ Similarly, co‐inheritance of α‐thalassemia is protective against some SCD‐related complications, such as hemolysis and stroke.^11^, ^13^, ^28^ However, most of the viable clinical expression remains unexplained by the genetic markers that have been examined. Indeed, availability of appropriate medical care, as in industrialized countries, usually mitigates morbidity and facilitates longer survival of people with SCD.^29^ In countries with less resources, on the other hand, morbidity is high and survival is low due to the combined effect of inadequate care and often severe clinical complications,^30^ compounded by other factors such as malnutrition, poverty, and exposure to pathogens.^13^, ^31^ Studies have also found that pain severity is associated with exposures to a variety of climate factors, including temperature fluctuation, wind speed, humidity, and high altitudes, as well as acute air influences such as air pollutants.^31^, ^32^, ^33^ Although these social and environmental influences on SCD complications are known, the exact mechanisms and pathways through which they influence clinical variability and phenotype are not well understood. Yet, there is limited research on the roles of these and other exogenous factors in SCD outcomes and even less published empirical research on the joint impacts of endogenous and exogenous factors. Major knowledge gaps also exist concerning the characteristics and effects of some dimensions of biology such as the microbiome and epigenome.

The scientific and social histories of SCD, coupled with its substantial individual and population variability, have led to the identification of SCD as an ideal model for elucidating mechanisms by which diverse endogenous and exogenous factors combine to influence health, disease, and health disparities.^10^, ^26^, ^27^ Along with spurring new pharmacological, genomic, and other cellular‐ or molecular‐level interventions, adequately resourced and well‐designed integrative SCD research could identify structural, social, environmental, and behavioral targets for prediction, prevention, treatment, and management of SCD complications that are more practical, safe, accessible, and affordable. Integrative SCD research could also complement and inform existing and emerging curative efforts such as bone marrow transplantation, gene therapy, and somatic cell genome editing.

## 3 ADVANCING INTEGRATIVE SICKLE CELL DISEASE RESEARCH

**FIGURE 1:**
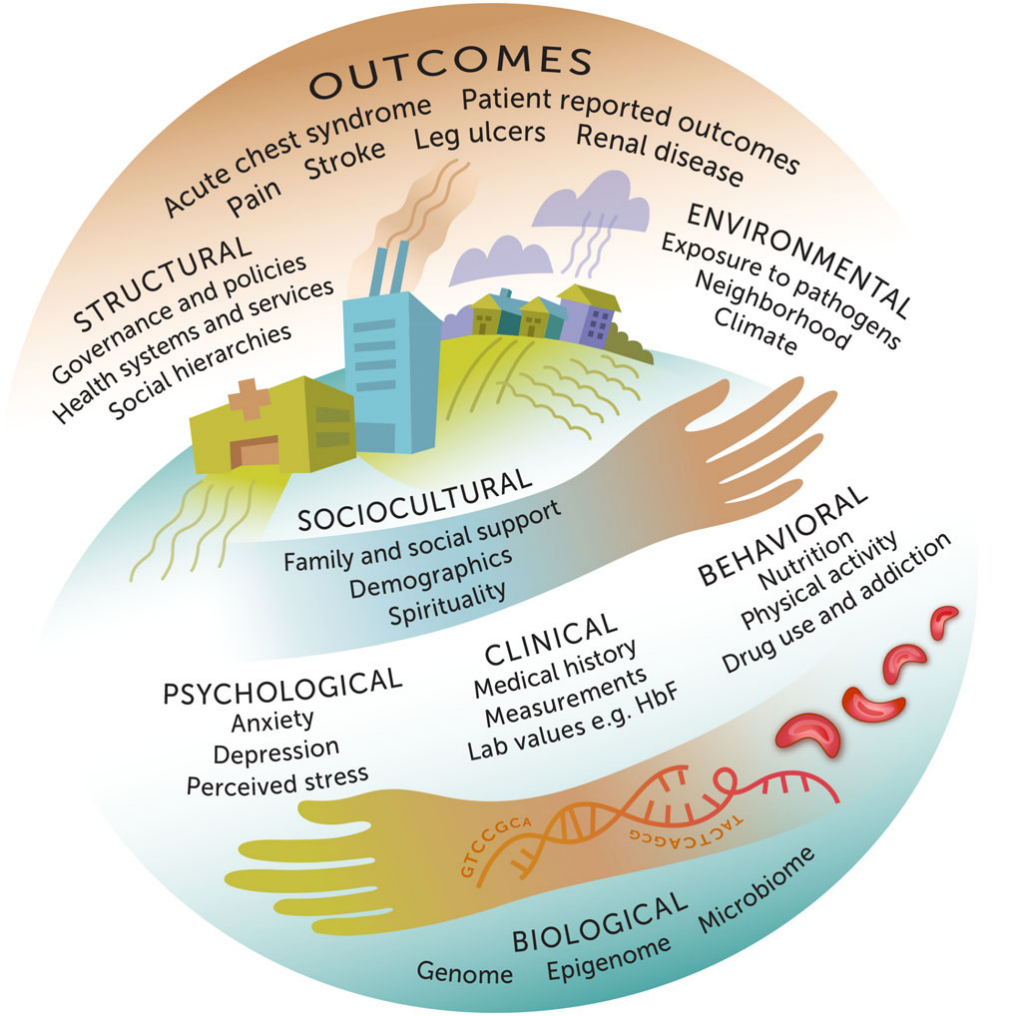
SCD theoretical framework. The spherical image symbolizes the global reach of sickle cell disease. The diverse disease‐related outcomes include both clinician reports and patient reports. The seven domains of influence—biological, psychological, clinical, behavioral, sociocultural, structural, and environmental—through their respective variables, comprise intricate networks that interact with one another over the life course. The absence of boundaries around each domain or the outcomes illustrates the interconnections and nonlinear relationships within and among them and the inherent challenges in distinguishing causes and effects. This non‐exhaustive representation of sickle cell disease ecology serves as a template for development and validation of an integrative conceptual model. Proof‐of‐concept studies using an array of modern exploratory data analytic techniques could produce a set of competing conceptual models that can in turn be tested statistically

We created an illustration that depicts the confluence of biological, psychological, clinical, behavioral, sociocultural, structural, and environmental factors/domains and SCD outcomes (physician‐ and patient‐reported) in a global context (Figure 1). This theoretical framework is guided by the Identity Networks, Genome, and Affect as Modulators of Health (INGAM) model,^26^ an integrative data‐driven model that attempts to clarify the degree of interdependence between social ecology and genomic processes. The INGAM model draws on principles from nonlinear dynamics, delineating major lines of bifurcation, in an effort to operationalize a multifactorial approach to health and illness. It facilitates understanding of not only the distal factors contributing to health conditions, but also the proximal mechanisms by which disease occurs or health can be restored and maintained. Consequently, INGAM opens the door for the exploration of more effective preventive and treatment measures, or for engineering new ways of delivering care to individuals and communities facing a number of daunting health problems.^26^

Figure 1 posits that the potential causal influence of a given factor, or node, in the system is sensitive to the state of the other nodes in the system. This sensitivity may, for example, take the form of mediating effects, multiplicative (interactive) effects, or feedback loops. For each of the seven “antecedent” domains in the framework, there are selected examples of variables linked in the extant literature to one or more of the SCD outcomes or to some measure of disease severity.^13^, ^34^, ^35^ Furthermore, variables in some of the “antecedent” domains, such as psychological and behavioral, can themselves be outcomes of the SCD experience. This reinforces the fluidity and complexity of the associations among and within the domains.

**FIGURE 2:**
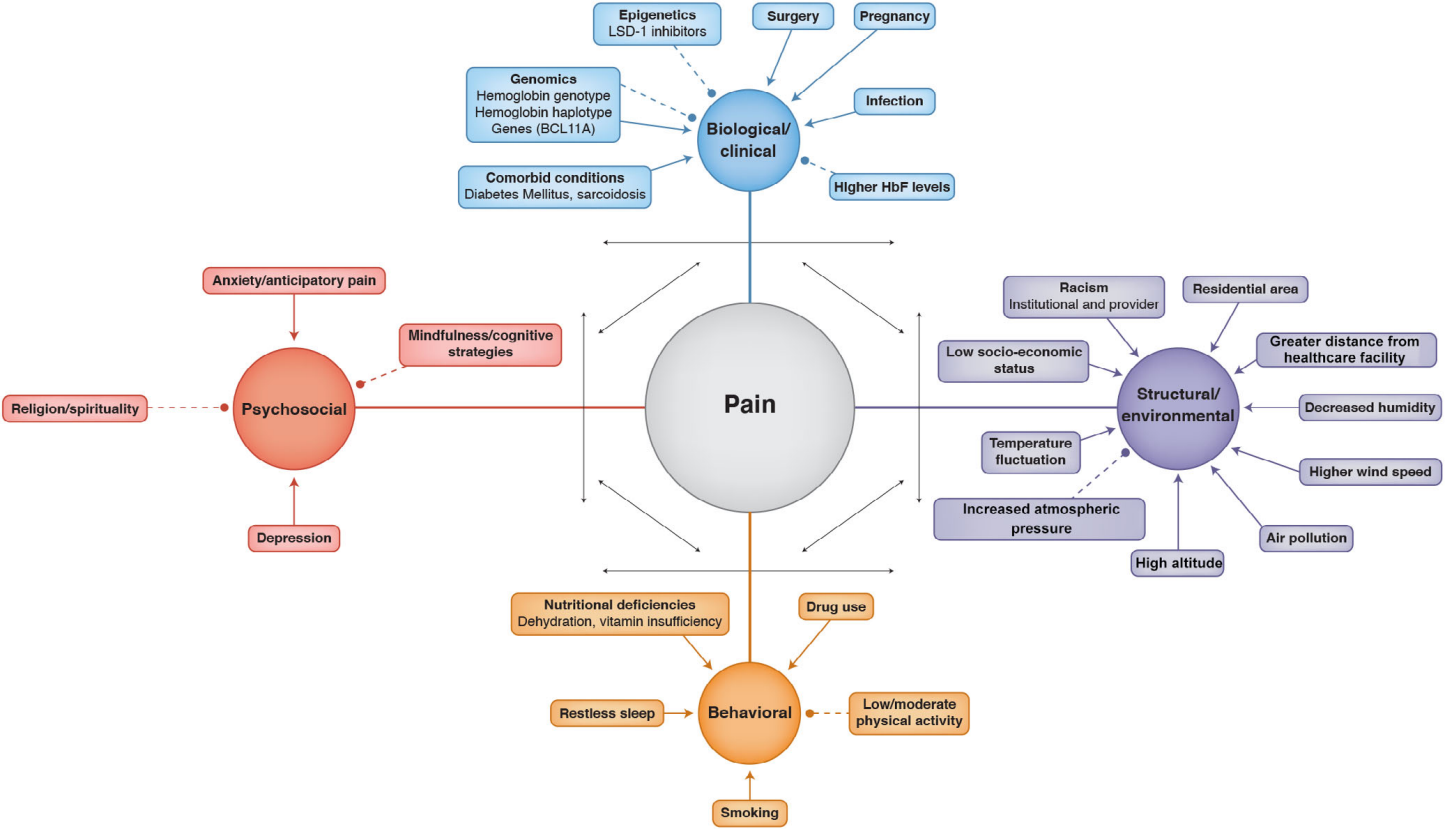
Preliminary conceptual model for SCD pain. Solid colored arrows indicate increased likelihood of pain or worsening prognosis. Dashed colored lines indicate factors that inhibit pain, or are beneficial by decreasing likelihood of pain occurrence. Double‐headed arrows indicate the interaction and interplay between domains (as in the examples of Psychosocial/Behavioral or Biological/Behavioral). Genomics (Biological/Clinical domain) has both solid colored arrows and dashed colored lines because specific genotypes, haplotypes, or genes are associated with either increases in severity of pain or reduction/mitigation of pain

Focusing on pain, the most common complication of SCD, we constructed a preliminary conceptual model (Figure 2) from our theoretical framework. Factors influencing SCD pain were synthesized from the literature and organized into 4 domain clusters: (1) Biological/Clinical,^12^, ^13^, ^28^, ^29^, ^30^, ^34^, ^35^, ^36^, ^37^, ^38^, ^39^ (2) Structural/Environmental,^13^, ^27^, ^31^, ^32^, ^33^, ^40^, ^41^ (3) Psychosocial,^42^, ^43^ and (4) Behavioral.^44^, ^45^, ^46^ Double‐headed arrows connecting the clusters indicate that all factors may display inter‐cluster relations. Although not displayed, factors within a cluster may also interact with one another. For example, nutritional deficiency, though classified as a behavioral attribute, is highly interlinked with structural/environmental factors such as residential area and socioeconomic status, biological/clinical factors such as genetics, and other behavioral factors such as drug use. Conceptualizations and frameworks of SCD tend to emphasize genetic and clinical contributors to various disease complications, including pain. Although genetic and genomic factors can influence the manifestation and experience of pain, a myriad external factors can modify or interact with an individual's genomic or clinical attributes, confer protection against impending pain episodes, or influence the severity and intensity of pain. While understanding an individual's biological or clinical history may grant clinicians some understanding of the physiological processes underlying pain episodes, it fails to capture the multidimensional nature of SCD. Subsequently, pharmacological treatment of pain may be a process to be included in a multifactorial management design that takes into account a patient's socioeconomic status, residential area, and mental health. Factors in Figure 2represented by solid colored arrows represent known contributors to pain or those that increase the likelihood of worsening pain. Dashed colored lines signify known mediators which lessen/prevent pain or confer protective effects. The mechanisms through which these factors act are also not well known and merit further research. LSD1 inhibitors^34^ and BCL11A silencing^39^ are included as examples of potential therapeutic mechanisms. A variety of protective factors, including HbF, coping mechanisms, and some behavioral factors may be critical targets for integrative research and interventions.

**FIGURE 3:**
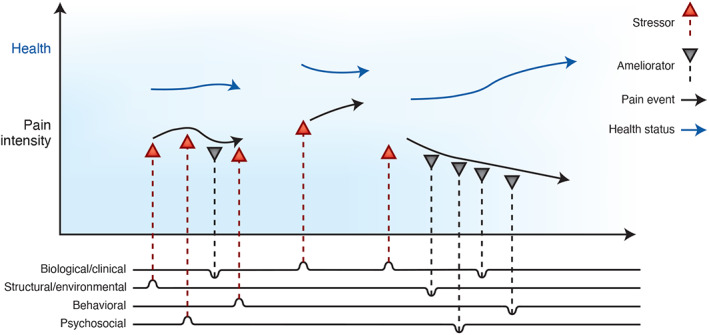
Conceptual health timeline for longitudinal analysis of contributors to pain. Solid blue arrows indicate the overall trajectory of health measured during a recorded outcome. Solid black arrows underneath mark intensity and duration of pain events. Factors from 4 domain clusters are represented by vertical dashed arrows under the graph where they drive the worsening or amelioration of pain events. Factors appearing under a pain event are thought of as associative, whereas factors that appear before an impending pain event signify causal or precipitative effects

Understanding how factors converge and precipitate or influence SCD complications longitudinally is critical in modeling the complexity of SCD and the processes through which phenotypic variations in outcomes such as pain are expressed.^47^ Temporal modeling of gene‐environment interactions in disease trajectory can be accomplished with tools such as observational data, medical records, and genomic/transcriptomic analysis.^47^, ^48^, ^49^ For SCD pain, modeling could be done through the creation of a “health timeline” (Figure 3), in which intensity, duration, and frequency of stressors and protective factors (see Figure 2) are measured and observed for an individual experiencing a recorded pain outcome. In this conceptual timeline, factors that occur before a pain episode would be classified as precipitating factors, whereas factors that occur during an episode would be considered associative. The frequency and duration of factor co‐occurrence as they correlate with pain events would provide an innovative method of quantifying the nature of co‐factor associations as well as factor/outcome relationships.

We initiated a study to explore and model relationships of sociodemographic, clinical, genetic, and environmental factors to pain among adults with SCD from three countries in Africa and the African Diaspora ‐ Cameroon, Jamaica, and the US. The pilot phase, which utilized existing patient data (N = 5314), was conducted between 2014 and 2016 by an interdisciplinary team of faculty, students, and research staff from the three countries and various fields such as medicine, nursing, human and medical genetics, genetic counseling, African & African American studies, cultural anthropology, global health, behavioral medicine/health psychology, statistics, epidemiology, and bioethics. The sizes and basic demographics of the study samples were as follows: Cameroon (Yaoundé Central Hospital), n = 264, median age = 24 years, 53% women; Jamaica (Sickle Cell Unit‐Caribbean Institute for Health Research), n = 4512, median age = 42 years, 51% women; and US (Duke University Hospital), n = 538, median age = 38 years, 57% women. The data from Cameroon were collected over a 3‐year period, while the data from Jamaica and the US spanned 5 years. Here we share some insights and preliminary findings from the pilot that could inform global SCD research agendas and guide future endeavors to develop innovative validated tools for understanding separate and joint contributions of multiple factors to phenotypic diversity in SCD.

Both the procedural and scientific aspects of the pilot project produced successes (eg, invaluable cross‐cultural teamwork and interdisciplinary collaboration) and challenges. Accessing and working with the data were by far the most trying tasks. Each institution had different requirements for the use of existing patient data, resulting in different amounts of time and effort for obtaining ethics approval. At two of the sites, logistical issues such as structure of the original consent prohibited sharing of the genetic data. Additionally, not all sites had electronic health records, and across sites there were different procedures for storing, sharing, and accessing data.

Our initial plan included applying accepted techniques for data harmonization.^50^, ^51^ After obtaining institutional research ethics approvals and retrieving the data, it was clear that there would be substantial barriers to achieving our aim of modeling relationships and making meaningful comparisons across sites. For example, datasets from two sites captured information from each separate clinic visit, while data from the other site were aggregated across clinic visits with only one summary value per patient for a given variable. Thus, we could not adequately compare all sites with respect to temporal associations among variables. There were also problems regarding poor standardization of variable names within the dataset at one site.

Ultimately, we elected to abandon any across‐site integration and instead conduct a few simple, largely descriptive analyses within each dataset. We judged that the data from Jamaica were in a condition most amenable to a reliable statistical analysis. Using these data, we estimated the associations between a set of environmental variables and pain episodes using a multivariable logistic regression model. The Huber‐White sandwich estimator was used to account for multiple visits per person (ie, within‐person clustering). Complete data for the variables under study were available from 4404 adults collected from 35 431 clinic visits over 5 years. The subsample had a mean age of 38 years, 55% of whom were women. We found that younger age, colder atmospheric temperature, higher rainfall, lower environmental zinc, and greater poverty were associated with a higher probability of a pain episode. There was no evidence that these associations differed across hemoglobin genotypes. Neither sex, urban vs rural location, elevation, nor geographic distance from a clinic exhibited any appreciable evidence of an association with the probability of a pain episode. Although several of the positive findings corroborate those from previous studies and have important implications for pain prevention and management,^31^, ^32^, ^33^ we caution that all of the pilot study findings (positive and negative) should only be interpreted as preliminary.

The pilot study confirms the importance and urgency of efforts to streamline policies and procedures for informed consent and the storage, sharing, and use of genomic and other types of data. Our initial work also underscores the need for comprehensive, valid, and sustainable standardization of SCD vocabulary and phenotyping. The SCD Ontology (SCDO) developed by an international group of SCD experts is a promising mechanism for meeting this need.^52^ Tools such as the SCDO are essential for large cohort studies and surveillance systems, whether utilizing secondary data or collecting primary data to avert some of the challenges with data harmonization. Experts in computational and statistical data sciences who can skillfully apply and generate novel tools and algorithms for high‐dimensional modeling and data analysis are vital to the interdisciplinary research teams needed to implement this potentially transformative work.

Establishment and deployment of a validated integrative model for SCD research could revolutionize our approaches to SCD and to health and illness in general, leading to more precise and effective methods for enhancing quality of life and reducing healthcare costs for individuals, families, communities, and populations worldwide.

## 4 CONCLUSION

The increasing attention to SCD is heartening and sets the stage for refinement of a common vision for the future of SCD and for global health. SCD is a natural laboratory for exploring perplexing questions at the intersection of human history, human variation, human identity, and human health. It offers a unique opportunity to broaden the repertoire and impact of precision medicine research and other 21st century integrative biomedical undertakings.

From evolution to genome editing, SCD is preeminent in research on both the origins and eradication of human disease.^11^, ^14^, ^53^ Whether the first molecular disease becomes the first to have an approved molecular cure from CRISPR remains to be seen. In the meantime, we must continue to scale up efforts to develop new tools and techniques for reducing and preventing complications in the millions of people worldwide suffering from SCD, most of whom are unlikely to have immediate access to high‐tech cures when they become available. We note that with SCD unmet needs are prevalent, even in European and North American countries.

Large national and multinational integrative studies are needed to better understand SCD globally and catalyze the development, translation, and implementation of locally‐appropriate interventions and policies. International organizations such as the WHO are best positioned to incentivize countries to adopt this integrated approach to SCD as a complex disease, just as the WHO urged countries in 2006 to develop and implement national programs for the management of SCD and countries in turn established clinical care guidelines for SCD.^21^, ^54^, ^55^, ^56^, ^57^ The recent SCD report from the US National Academies of Sciences, Engineering, and Medicine could serve as a comprehensive roadmap for the WHO and other organizations seeking to encourage or fund ecological SCD research that will accelerate improved health and healthcare at local, national, and global levels.^58^ Although this work is challenging and requires ample resources, it will yield tremendous rewards for human knowledge and wellbeing far beyond SCD.

## CONFLICT OF INTEREST

Charmaine DM Royal has nothing to disclose. Michael Babyak has nothing to disclose. Nirmish Shah reports personal fees from Novartis, outside the submitted work. Shantanu Srivatsa has nothing to disclose. Kearsley A. Stewart has nothing to disclose. Paula Tanabe reports grants from AHRQ, grants from NHLBI, outside the submitted work. Ambroise Wonkam has no conflict of interest to declare. Monika Asnani has nothing to disclose.

## AUTHOR CONTRIBUTIONS

Authors were part of the team that led the pilot study described in the paper. Michael Babyak compiled, managed, and analyzed the existing data provided by Monika Asnani, Ambroise Wonkam, and Nirmish Shah for the pilot. Charmaine DM Royal conceived the manuscript and wrote the first draft, with contributions from Monika Asnani, Michael Babyak, and Shantanu Srivatsa. All authors have read and provided intellectual comments on the draft, edited it for key content, and approved the final version of the paper.
